# Housing the Nursing Staff

**Published:** 1920-12-18

**Authors:** Gladys M. E. Leigh


*To the Editor of* The Hospital.

### Housing the Nursing Staff.

*To the Editor of* The Hospital.

Sir,—In the columns of the current issue of The Hospital we are told that the lack of sleeping accommodation for the nursing staff of the North Staffordshire Cripples' Hospital is so deplorable that some of the nurses are sleeping in three huts in the garden, and *one of the nurses in a loft.* If this statement is literally correct, surely it is time there was a public inquiry into the administration of a hospital that allows its nurses to live under such conditions. Are the people of Staffordshire content that the women who are nursing the cripples of their county shall be housed in so deplorable a manner, and what provision do they propose to make for these women if their health breaks down as a result of the aforesaid conditions?

Are the House Committee, the Medical Board, *and the Matron* satisfied "to carry on" in this way?—Yours faithfully,

Gladys M. E. Leigh.

